# Recent progress in the structure control of Pd–Ru bimetallic nanomaterials

**DOI:** 10.1080/14686996.2016.1221727

**Published:** 2016-09-19

**Authors:** Dongshuang Wu, Kohei Kusada, Hiroshi Kitagawa

**Affiliations:** ^a^ Division of Chemistry, Graduate School of Science, Kyoto University, Kyoto, Japan

**Keywords:** Palladium, ruthenium, nanomaterials, alloy, catalysis, 60 New topics/Others, 106 Metallic materials, 205 Catalyst / Photocatalyst / Photosynthesis, 301 Chemical syntheses / processing

## Abstract

Pd and Ru are two key elements of the platinum-group metals that are invaluable to areas such as catalysis and energy storage/transfer. To maximize the potential of the Pd and Ru elements, significant effort has been devoted to synthesizing Pd–Ru bimetallic materials. However, most of the reports dealing with this subject describe phase-separated structures such as near-surface alloys and physical mixtures of monometallic nanoparticles (NPs). Pd–Ru alloys with homogenous structure and arbitrary metallic ratio are highly desired for basic scientific research and commercial material design. In the past several years, with the development of nanoscience, Pd–Ru bimetallic alloys with different architectures including heterostructure, core-shell structure and solid-solution alloy were successfully synthesized. In particular, we have now reached the stage of being able to obtain Pd–Ru solid-solution alloy NPs over the whole composition range. These Pd–Ru bimetallic alloys are better catalysts than their parent metal NPs in many catalytic reactions, because the electronic structures of Pd and Ru are modified by alloying. In this review, we describe the recent development in the structure control of Pd–Ru bimetallic nanomaterials. Aiming for a better understanding of the synthesis strategies, some fundamental details including fabrication methods and formation mechanisms are discussed. We stress that the modification of electronic structure, originating from different nanoscale geometry and chemical composition, profoundly affects material properties. Finally, we discuss open issues in this field.

## Graphical abstract

**Figure:**
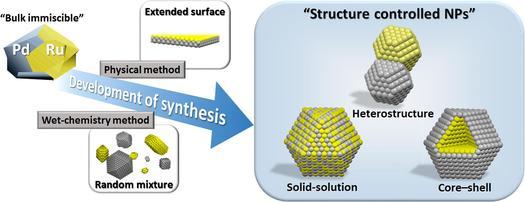

## 1. Introduction

Pd and Ru, which, respectively, adopt the face-centered cubic (fcc) and hexagonal close-packed (hcp) structures in bulk, are two key elements of the Pt group metals closely related to our society. Pd is invaluable to many industrial reaction processes such as hydrogenation/dehydrogenation reactions,[1] purification of automotive pollutants,[2] low-temperature fuel cell reactions,[3] C–C bond formation and C–H bond activation.[4] In addition, Pd is also a key element for ‘hydrogen economy’.[5] Ru and its compounds are also in a class of particularly significant catalysts for many reactions, for instance, ammonia synthesis,[6] C–N formation,[7] hydrogenation reaction [8] and oxygen evolution reaction.[9] As a nonmagnetic conductor, Ru is an indispensable part of spintronics, which is used to separate two magnetic layers.[10] Recently, Ru has attracted much attention as a catalyst for CO oxidation.[11] Although Pd and Ru have their own unique properties, they show similar effects for many applications, e.g. CO oxidation and some organic reactions.[11,12] Therefore, naturally, it is interesting to develop Pd–Ru bimetallic materials, in which much improved or novel properties are expected.

**Figure 1.**
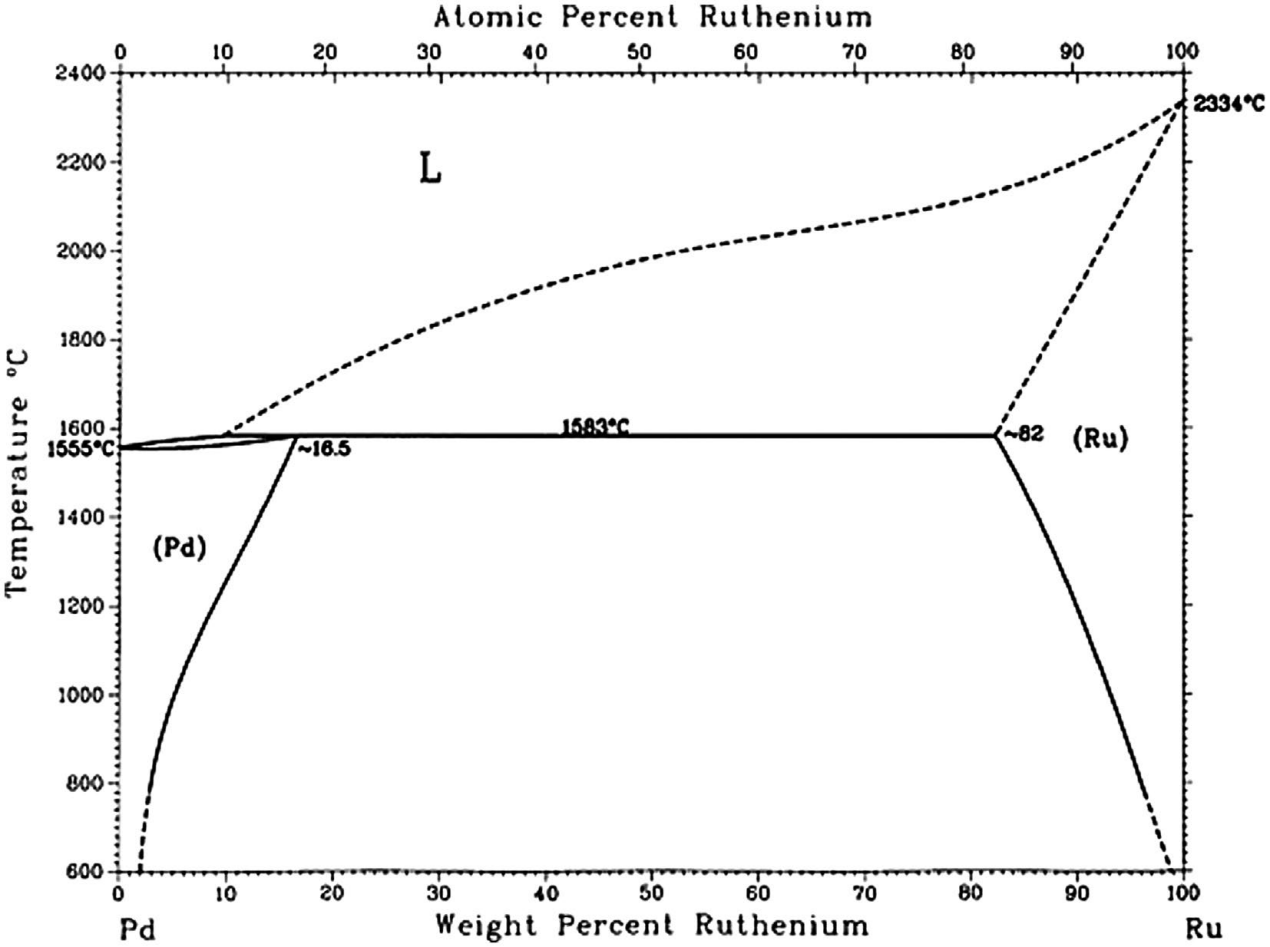
The Pd–Ru equilibrium bulk phase diagram. (Reproduced with permission from [32] © 1993 Springer.)

As early as 1966, Pd–Ru bimetallic alloys were used as hydrogen diffusion membrane in a US patent filed by Engelhard Industries Corporation,[13] motivated by the study of hydrogen storage/diffusion properties of Pd and Pd-based bulk alloys.[14–20] In this patent, Pd–Ru bulk alloy membranes containing 1–10 wt% Ru content were prepared by high temperature annealing. The patent claimed that Pd–4.5%Ru bulk alloy membrane had higher hydrogen permeability than pure Pd and Pd–Ag bulk alloy. Additionally, the patent reported that the Pd–Ru alloy membrane had much higher tensile strength than that of Pd after being annealed at high temperature. Such high tensile strength of Pd–Ru alloy membrane had also been supported by other research groups such as Cabrera and co-workers in 1995 [21] and Gade et al. in 2009.[22] However, years later, contradictory observations on the permeability of Pd–Ru alloy were reported. Cabrera et al. [23] studied the kinetics of hydrogen desorption Pd and Pd–Ru bulk alloy foils^23^. The results indicated less solubility of hydrogen in the Pd–5%Ru alloy than pure Pd. Moreover, the diffusivity of hydrogen in the Pd–5%Ru alloy was slower than in pure Pd. The activation energy for bulk diffusion was 4.9 kcal mol^–1^ for Pd–5%Ru alloy, which was higher than that of pure Pd (4.4 kcal mol^–1^). The decrease in hydrogen absorption capacity for Ru-Pd alloy was further studied by hydrogen electrosorption and thermal programmed desorption.[24,25] It is known that the hydrogen absorption/desorption is extremely dependent on the structure of Pd–Ru samples, such as Ru composition and film thickness. Therefore, a precise characterization of the Pd–Ru alloy is needed to confirm the hydrogen absorption/desorption properties. Since 1966, the Pd–Ru bulk alloy system has been widely studied in areas including hydrogen storage/permeability,[22,25,26] selective hydrogenation[27–30] and magnetic properties.[31] These studies pointed out the alloy effect by substituting a low amount of Ru with Pd caused the modification of the Pd d-band electronic structure and consequently changed the Pd properties. However, in this stage, most of the Pd–Ru bulk alloy were obtained by techniques such as conventional cold rolling, physical/chemical vapor deposition, arc melting, electroplating or electroformation and electroless plating,[27] and the Ru content of the alloys was generally limited below 10 wt%. In fact, it is difficult to obtain Pd–Ru alloys with arbitrary metallic ratio in bulk phase, because Pd and Ru cannot mix each other more than around 15% even near the melting point of Pd, as shown in the equilibrium phase diagram (Figure 1).[32]

In addition to composition control, to maximize the potential of Pd and Ru elements, a significant effort should be devoted to controlling the structure of Pd–Ru bimetallic materials. The properties of materials highly depend on their structure. For instance, Alayoglu and Eichhorn found that Rh@Pt and RhPt solid-solution alloy NPs show different catalytic behavior towards CO oxidation.[33] Such differences were attributed to the distinct electronic structure and surface atomic arrangement on different structures. Therefore, it is highly desirable to synthesize Pd–Ru bimetallic alloys with a suitable structure, which can fully bring out their potentials for target applications. However, a rational control of the structure of Pd–Ru bimetallic alloy is difficult because of the limited solubility between Pd and Ru,[32] compared with all proportional solid-solution system such as Rh-Pt system.

Due to the explosive growth of nanoscience and nanotechnology, we are now capable of designing and constructing alloy NPs with a wide range of combinations and compositions. In general, when particle size decreases, the corresponding surface energy increases and the Gibbs free energy of the mixture decreases, causing deviations from the phase diagram of the bulk metals.[34] Thus, some solid-solution alloys consisting of two bulk immiscible metals have been obtained at the nanoscale, e.g. Fe–Ag[35] and Ag–Rh [36] alloy NPs. These newly obtained nanostructures may show novel properties that are not exhibited in the bulk phase. For example, our group found that Ag_0.5_Rh_0.5_ solid-solution NPs can absorb hydrogen, though bulk Ag and Rh cannot absorb hydrogen at all.[36]

During the past two decades, synthetic methods of bimetallic nanomaterials have been established and we are now able to control their structure including solid-solution alloys, core–shell and heterostructure in some alloy systems.[37–39] To date the structures of several types of Pd– or Ru–based bimetallic nanomaterials, for instance, Pd–Pt,[5,40–42] Ru–Ni,[43,44] and Ru–Pt [45–47] bimetallic nanomaterials with core–shell structure, heterostructure or solid-solution alloy have been successfully controlled. However, the nanostructure-controllable synthesis of the Pd–Ru bimetallic system is rarely reported. According to theoretical calculations, the bond energy of Pd–Ru (0.442 eV) is much higher than that of other noble metal M–Ru bonds, such as Pt–Ru (0.297 eV) or Rh–Ru (–0.012 eV).[46] In the early stage, only a physical mixture of Pd and Ru NPs was obtained by impregnation or high-temperature gas reduction methods.[48,49] However, even such a physical mixture exhibited better properties than the NPs of the pure metal parents. Following, many groups have reported Pd–Ru phase-separated alloy NPs and solid-solution random alloy NPs with the progress of wet-chemical methods such as polyol and solvothermal methods, although the structures in most of these reports were less evidenced. Very recently, our group successfully synthesized and characterized the Pd–Ru solid-solution alloy NPs within the whole composition range.[50] Since then, there have been successive reports on the syntheses of well structure-controlled Pd–Ru bimetallic NPs including core–shell structure,[46,51] a heterostructure,[52] and solid-solution alloy NPs,[50,53,54] and the corresponding applications.

In this review, we focus on the recent development of Pd–Ru bimetallic nanomaterials including the synthetic methods, as well as the corresponding applications. At the beginning, we will briefly introduce the NSAs and the physical mixture of Pd and Ru NPs, in which a synergistic effect was found between the Pd and Ru elements. Next, we will review the Pd–Ru bimetallic nanomaterials from two aspects according to the mixing patterns of the atoms. One aspect is the phase-separated nanoalloys, including heterostructure and core-shell structure. The other is solid-solution alloy NPs. In combination with some advanced analytical tools, we will discuss how the structure and composition affect the electronic structure at the atomic level, and finally the corresponding properties. Finally, some open issues in this field are proposed.

## 2. Near-surface alloys

NSAs, defined as alloys wherein a solute metal is present near the surface of a host metal in a concentration that is different from the bulk, may form even when the bulk alloys are not thermodynamically stable.[55] As a model system, NSAs provide clear atomic-level structural information related to how the electronic states of Pd and Ru vary by changing the atomic arrangement at the interface region. In the 1990s, with the development of surface science, ultra-high vacuum technology, and theoretical calculation, the NSAs of Pd and Ru were readily prepared and studied. Several research groups, such as Ross’s [56] and Goodman’s [57] prepared Pd monolayer and/or multiple layers pseudomorphically grown on an Ru (0001) surface by electron-beam evaporation and investigated the core-level shift of Pd 3d binding energy with X-ray photoelectron spectroscopy (XPS). They found that the core-level spectra of Pd 3d positively shifted, indicating that the d-band center of the valence band moved away from the Fermi level in comparison with bulk Pd (111). As a result, pseudomorphically grown Pd thin film on a Ru (0001) surface showed a weaker CO adsorption energy than the Pd (111) and Ru (0001) surfaces.[58] Such experimental results were consistent with Hammer and Nørskov’s [59,60]density functional theory (DFT) calculations on the relationship between d-band center and catalytic properties, i.e. the well-known ‘d-band center theory’.

Recently, more advanced analytical tools in combination with theoretical calculations are being adopted to promote our understanding of the Pd–Ru bimetallic nanomaterials. For example, Behm’s group studied the stability and tendency of segregation of Pd–Ru/Ru (0001) surface alloys under high-temperature annealing by scanning tunneling microscopy (STM) and Auger electron spectroscopy (AES).[61] One or two layers of Ru atoms were deposited on the top of equilibrated Pd–Ru NSAs with certain metal ratios. It was found that the Pd atoms in the sublayer would migrate to the outermost layer to form a surface alloy after annealing. The newly formed surface alloy had the same lateral metallic distribution with the initial equilibrated surface alloys before overgrowth by Ru. Such ‘floating back properties’ indicated that surface alloys represented stable surface configurations.[61] Ramos et al. [62] investigated the dissociative adsorption of molecular hydrogen on Pd_x_Ru_1-x_/Ru (0001) (0 < x < 1) by means of He atom scattering, DFT and quasi-classical trajectory calculations^62^ and found that in a Pd-rich surface alloy the reactivity of Ru atoms in dissociative adsorption of molecular hydrogen was enhanced by the presence of nearest neighbor Pd atoms. However, Pd atoms in the Pd-rich surface alloy were less reactive than the Ru ones regardless of their surroundings because of both electronic and strain effects. These examples undoubtedly showed that, even if just in the interfacial area, the chemical and catalytic properties of Pd and Ru were affected by the electronic states variation by forming Pd–Ru metal–metal bond.

## 3. Physical mixture of Pd and Ru NPs

Before the appearance of Pd–Ru nanoalloys, many groups adopted a physical mixture of monometallic Pd and Ru NPs for various applications. They found that even the physical mixture performed better than the corresponding monometallic NPs towards some applications. For example, Romanenko et al. studied the role of Ru additives in the stabilization of carbon-supported Pd NPs at high temperature in a hydrogen atmosphere.[48] The carbon-supported Pd–Ru catalysts were prepared by simultaneously spraying solutions of Na_2_CO_3_ and metal complexes (H_2_PdCl_4_ and/or Ru(OH)Cl_3_) in the presence of the carbon granules. After drying in a vacuum oven, gas-phase reduction with hydrogen was conducted at 250 °C and a mixture of Pd and Ru NPs was obtained. The Pd–Ru bimetallic catalyst showed enhanced durability at high temperatures in a hydrogen atmosphere (250–650 °C) and during terephthalic acid hydropurification (250–285 °C). They found that the carbon-supported Ru NPs maintained a high dispersity in these conditions. Although the dispersity of the Pd–Ru bimetallic catalyst decreased, it was better than that of pure Pd NPs. The authors did not find any change in the lattice constants of the Ru and Pd phases in such a physical mixture. Therefore, the chemical interactions between Pd and Ru were not the reason for the stabilization of the Pd catalysts. They considered that the highly sintering-resistant Ru NPs suppressed the migration of Pd.

Monyoncho et al. [49] prepared carbon-supported Pd–Ru NPs using a polyol method as an anodic catalyst for ethanol oxidation reaction (EOR). Metal precursor salts (PdCl_2_ and RuCl_3_) were dissolved in ethylene glycol (EG). After adjusting the solution pH to 8 by NaOH, the mixture was heated from room temperature (r.t.) to 160 °C to generate colloidal NPs. Although both X-ray diffraction (XRD) and XPS results indicated that Ru existed as a separated amorphous Ru oxide, Pd_90_Ru_10_/C and Pd_99_Ru_1_/C were found to be the best catalyst systems that exhibited more than four times higher mass activity (current density per mass of Pd) compared to pure Pd at –0.96 and –0.67 V versus MSE (mercury-mercurous sulfate electrode), respectively. In addition, the Pd–Ru catalysts showed lower surface deactivation from the EOR intermediates/products. The authors ascribed this phenomenon to the synergetic effect between the surface oxide species (PdO_x_ and RuO_x_), which lowered the EOR potential of Pd NPs. Only separated Pd NPs and a mixture of Ru amorphous oxides were obtained by heating the solution from r.t. to 160 °C in [18], although co-reduction in polyol solvent is a popular method for generating bimetallic solid-solution alloys. Similar synthesis conditions can be seen elsewhere, for example, co-reduction of metal precursors by NaBH_4_.[63,64] Such results point to the challenge in the synthesis of Pd–Ru solid-solution NPs.

## 4. Phase-separated nanoalloy: heterostructure and core-shell structure

**Figure 2.**
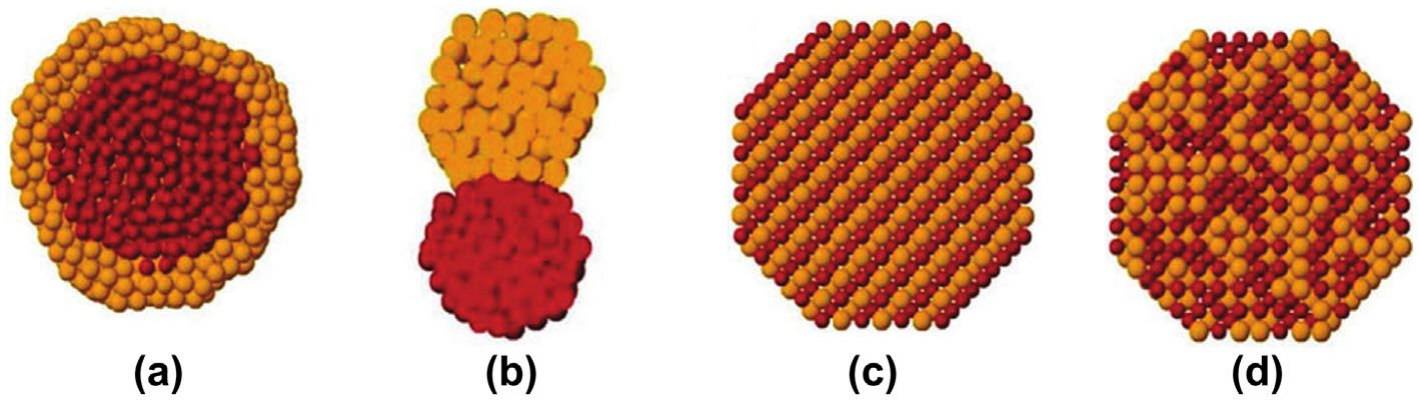
Illustration of a possible mixing pattern in a bimetallic alloy system. (a) the core–shell structure and (b) heterostructure belong to a phase-separated alloy. Solid-solution alloys include (c) an ordered alloy with long-range order and (d) random alloys with short-range order. (Reproduced with permission from [39] © 2012, Royal Society of Chemistry.)

Generally, nanoalloys can be classified into two types according to their mixing pattern, i.e. phase-separated and solid-solution.[39] Phase-separated alloys include core–shell structures and heterostructures (Figure 2(a) and 2(b)). The structural difference between a core–shell structure and heterostructure can be simply distinguished by the shared interface. For a core-shell structure, the secondary element covers the whole surface of the core element. For heterostructure, the constituent elements only share specific facets or sites. Solid-solution alloys are also of two types. One is a random alloy with random atomic order, and the other is an ordered alloy, which features long-range atomic order (Figure 2(c) and 2(d)). These structural differences can be determined by the powder XRD method. For core-shell or heterostructure alloys, the characteristic diffraction patterns of the parent metals appear together, whereas for solid-solution type alloys, only the Bragg diffraction pattern of the alloy itself is observed, without any diffraction patterns of the parent metals.[65]

For phase-separated nanoalloys, irrespective of whether they have heterostructure or core-shell structure, the formation process follows two steps. First, one element preferentially forms seeds/NPs, and then the secondary element attaches on the preformed seeds/NPs. According to the redox potential (Pd^2+^/Pd, 0.951 V *vs.* Ru^3+^/Ru, 0.386 V),[66] the Pd ion usually tends to be reduced much earlier and faster than the Ru ion in wet-chemical synthesis. Furthermore, the Ru and Pd elements have different bulk crystal structures. Defects or distortions are introduced at the interface between the fcc and hcp phases to decrease the surface energy, and, thus, epitaxial growth is considered difficult. Usually, the nucleation and growth processes of secondary elements are believed to be initiated on the facets of preformed seeds or NPs with the highest surface energy and then proceed to other facets with lower energies.[67] If the reduction rate is low, such as at a lower temperature, the nucleation and diffusion processes take place under equilibrium conditions, resulting in core-shell NPs or heterostructure. However, the reduction of Ru usually occurs at a higher temperature than for Pd. At high temperature, the fast reduction breaks down near-equilibrium conditions and favors the formation of alloy NPs by quick diffusion. Therefore, it is still a challenge to form uniform heterostructures and/or core-shell structures.

Before introducing the uniform Pd–Ru heterostructure and core-shell structure, we would like to introduce two interesting reports related to the shape-control of Pd using Ru ions. In one of these reports, Cao’s group [68] synthesized {111}-faceted Pd truncated triangular bipyramids (TTBPs) in a polyol process with the assistance of RuCl_3_ ^68^. The addition of RuCl_3_ can greatly promote the formation of single-twinned seeds of Pd. During the synthesis, RuCl_3_ was first reduced to Ru atoms, and the reduced Ru atoms were quickly consumed into positively charged Ru ions by a galvanic replacement (GR) reaction with PdCl_4_^2−^ in the solution. A trace amount of the Ru^2+^ species (around 0.6 at.%) was detected on the surface of TTBPs by XPS and Fourier transform infrared (FTIR) spectroscopy, verifying the proposed formation mechanism of TTBPs. Xiong’s group reported another example in 2015.[69] They simply introduced a certain amount of RuCl_3_ into the well-established protocol for Pd nanocubes by Xia et al.[70] surprisingly, well-defined concave Pd NPs with sizes up to 40 nm were obtained. The authors proposed that the mechanism should be a combination of an under-potential deposition (UPD) process of Ru atoms and subsequently a GR reaction between Ru atoms and PdCl_4_^2−^. Unlike the previous report of Cao’s group,[68] although small amounts of Ru atoms were detected in the intermediates, they gradually diminished during the Pd growth process. In [45] and [46] only trace amounts of Ru content were detected in the final product. The low temperature of the synthesis (100 and 80 °C, in the former and latter cases, respectively) might be the reason for very low Ru content. The reduction kinetics of Ru was too slow under such low temperatures. However, we think that the synthetic processes in the two reports are very interesting, because they indicated the appearance of an Ru-on-Pd structure as an intermediate, showing the possibility of obtaining a heterostructure and/or a core-shell structure.

### 4.1. Heterostructure between Pd and Ru

Heterostructure is an advantageous architecture for various applications such as p-n junctions. In particular, for a heterostructure constructed by two metals, fascinating properties are expected because of the modified electronic state in the interface region. As evidenced by the NSAs, the core-level binding energy of pseudomorphic Pd adlayers shifted to the higher energy side compared with bulk Pd.[56,57] Therefore, it is interesting to construct a uniform Pd–Ru heterostructure in a nanoalloy fashion.

**Figure 3.**
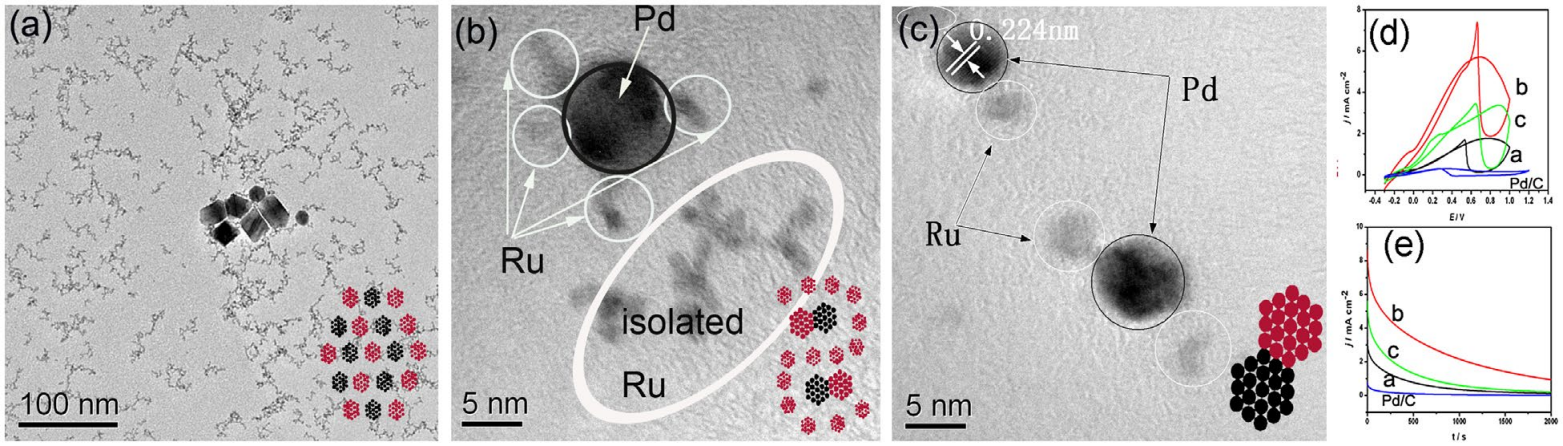
TEM images of (a) physical mixture composed of Pd polyhedrons and Ru NPs at 110 °C. (b) Mixed-phase containing both Pd–Ru nanodendrites and isolated Ru NPs prepared at 140 °C. (c) Heterostructure, Pd–Ru nanodendrites obtained at 170 °C. Insets in each TEM image are the models of the corresponding structures. Red and black represent Pd and Ru atoms, respectively. (d) and (e) Cyclic voltammetry (CV) and chronoamperometry (CA) behavior of the as-prepared Pd–Ru bimetallic systems and commercial Pd/C catalysts. (Reproduced with permission from [52] © 2012, Royal Society of Chemistry.)

To date, only one report clearly demonstrates the Pd–Ru heterostructure. In 2012, Wu et al. [52] reported on the controlled synthesis of Pd_0.5_Ru_0.5_ bimetallic nanomaterials by reducing the metal precursors in EG at different temperatures^52^. A single monometallic NP mixture consisting of polyhedron Pd and worm-like Ru NPs formed at 110 °C (Figure 3(a)). At 140 °C, the as-obtained product contained Pd polyhedrons, worm-like Ru NPs and a small amount of Pd–Ru nanodendrites (Figure 3(b)). A well-dispersed heterostructure of Pd–Ru nanodendrites formed at 170 °C (Figure 3(c)). All these Pd–Ru combinations showed higher catalytic activity and stability towards formic acid electrooxidation than the commercial Pd catalysts (Figure 3(d)), indicating a synergistic effect between Pd and Ru regardless of the different structural configurations. Interestingly, the sample with mixed-phase (140 °C) rather than the single-phase Pd–Ru nanodendrites (170 °C) was the best catalyst. Cyclic voltammetry (CV) indicated that the RuO_x_H_Y_ species generated during the scan might greatly enhance the catalytic activity and stability of Pd NPs. In addition, the separated Ru NPs in the mixed-phase sample (140 °C) may prevent the Pd NPs from aggregating.

Additionally, heterostructure has been reported as an intermediate during the preparation of other types of nanoalloys. For instance, during the preparation of sub-5 nm Pd–Ru solid-solution alloys by Wu et al., [53] Pd-on-Ru heterostructure with an average diameter about 3.2 nm was detected by high-angle annular dark-field scanning transmission electron microscopy (HAADF-STEM) as an intermediate^53^. However, with the processing of the reaction at high temperature, this Pd-on-Ru heterostructure gradually disappeared and finally turned into a solid-solution type structure. Based on these early reports, although well-defined Pd–Ru heterostructures have not yet been well studied, they are very likely to develop in the near future by careful design and control of nucleation and growth kinetics.

### 4.2. Core-shell Pd–Ru NPs

**Figure 4.**
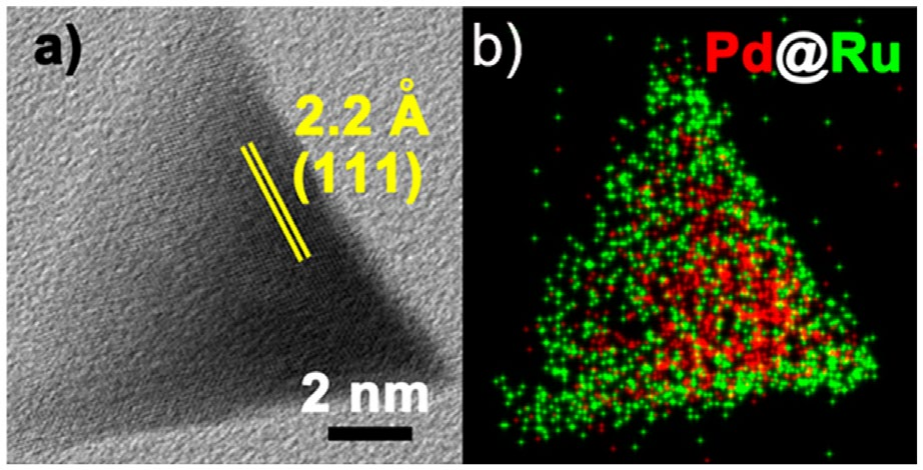
(a) HRTEM image and (b) EDX mapping of a single Pd@Ru tetrahedron. Red and green regions represent Pd and Ru, respectively. (Reproduced with permission from [46] © 2015, American Chemical Society.)

In 2015, Gu and co-authors [46] reported the synthesis of Pd@Ru structures via a two-step seeded growth process using the thermal solvent method^46^. They first synthesized Pd nanocrystals by a hydrothermal method. After collecting the as-prepared Pd nanocrystals, they added the Pd nanocrystals into the reaction solution containing a Ru precursor and triggered the epitaxial growth of Ru on preformed Pd nanocrystals. The core-shell nature was confirmed by energy-dispersive X-ray (EDX) mapping (Figure 4). The XRD patterns indicated that most of the Ru shells adopted the hcp structure. However, according to the authors, some high-resolution transmission electron microscopy (HRTEM) images showed that the Ru shells were incomplete in some Pd@Ru nanocrystals and a part of Pd surface was exposed. Interestingly, Rh–Ru solid-solution type nanoalloys were obtained under the same preparation process. Core-shell Pt@Ru NPs can be obtained either by a one-pot reaction or two-step seeded growth. In contrast, only Pd and Ru NPs physical mixture was obtained in a one-pot reaction. Such distinct results may originate from the different bond formation energy among Pd–Ru, Rh–Ru, and Pt–Ru metal–metal bonds.

**Figure 5.**
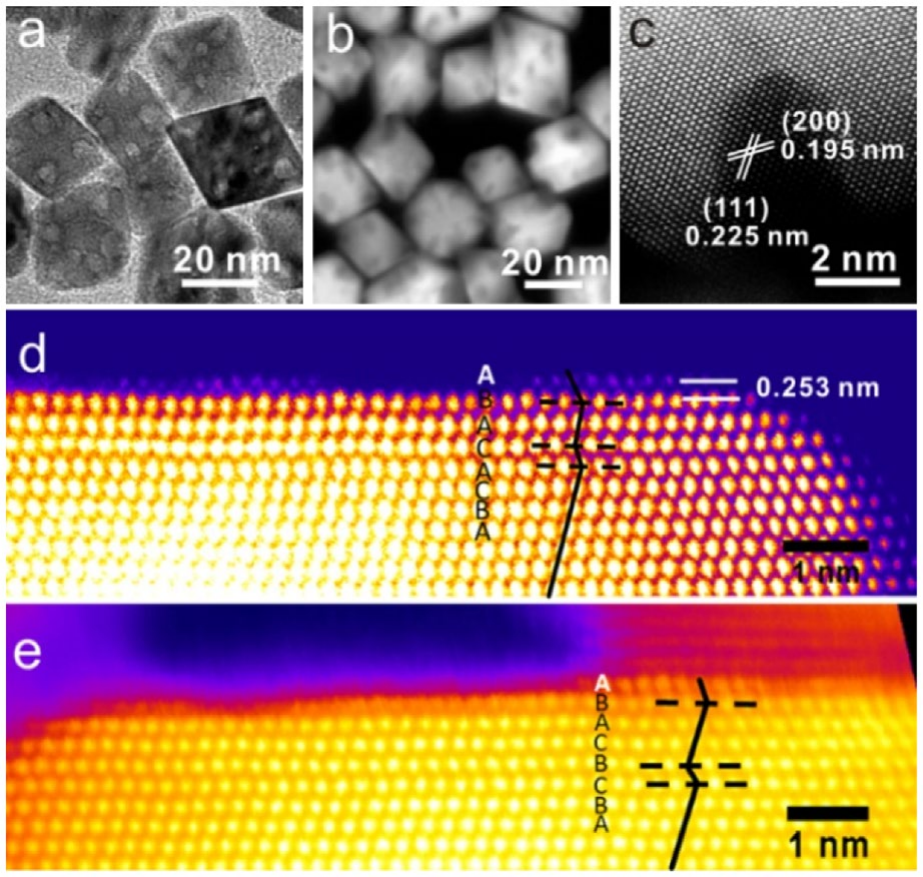
(a) TEM and (b) HAADF-STEM images of ordered porous octahedral nanostructures. Atomic resolution aberration-corrected HAADF-STEM images of (c) the porous area of the ordered porous octahedral nanostructure and (d, e) the surface of octahedra. False color was applied to enhance the contrast. (Reproduced with permission from [51] © 2015, American Chemical Society.)

**Figure 6.**
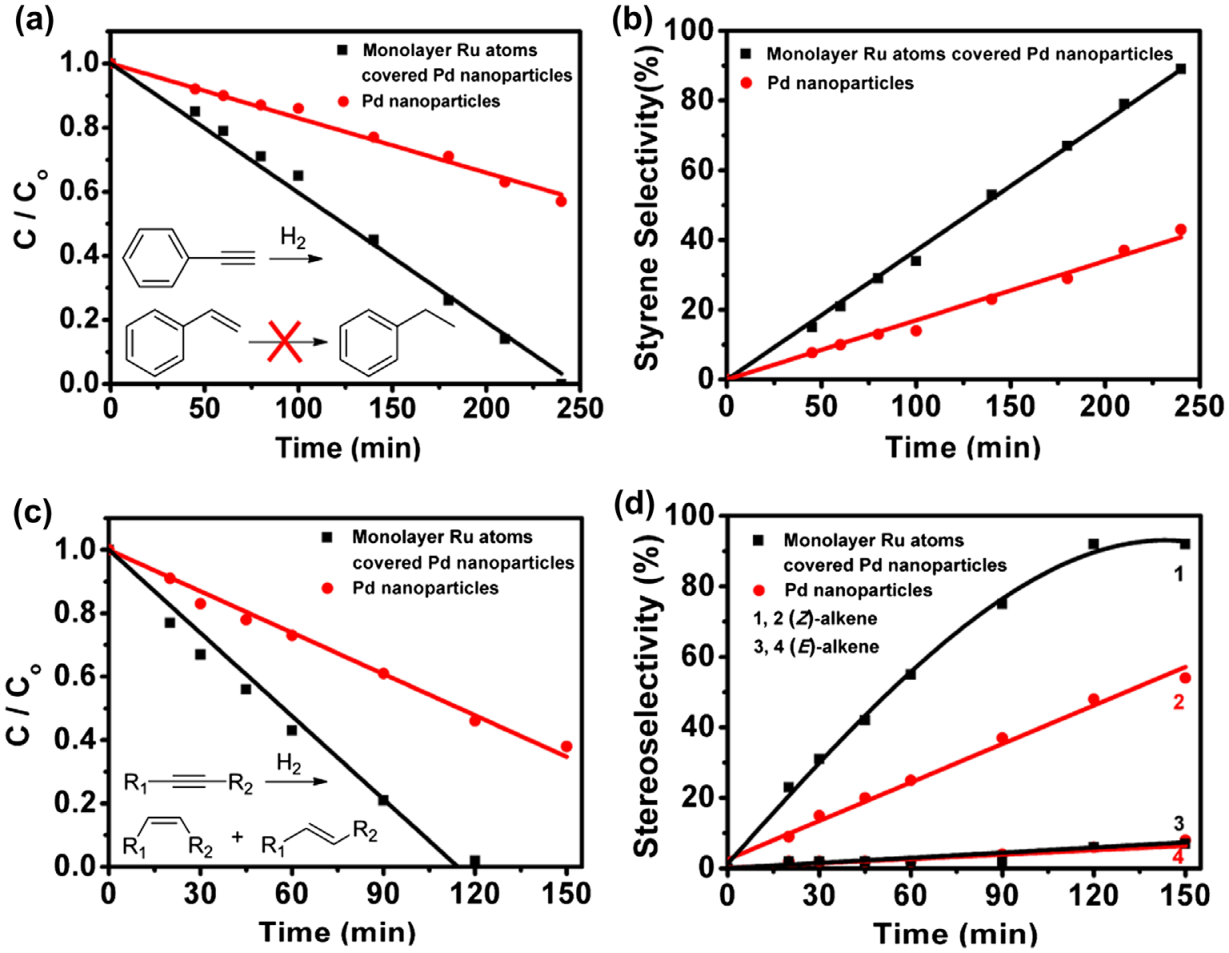
Catalytic semihydrogenation of alkynes. (a) Conversion of phenylacetylene and (b) selectivity of styrene catalyzed by the porous Pd octahedra covered with monolayer Ru atomic NPs and Pd NPs. (c) Conversion of methyl non-2-ynoate (R1 = C_6_H_13_, R2 = CO_2_Me) to (d) (Z)-methyl non-2-enoate (curves 1 and 2) and (E)-methyl non-2-enoate (curves 3 and 4) catalyzed by porous Pd octahedra covered with monolayer Ru atoms NPs and Pd NPs. (Reproduced with permission from [51] © 2015, American Chemical Society.)

In a recent report, Li’s group [51] synthesized highly ordered porous Pd octahedrons covered with a Ru monolayer in one-pot reaction^51^. A combination of the hydrothermal method and a UPD process yielded the deposition of Ru {001} monolayer on the surface of Pd {111} facets. In fcc metals, an octahedron single-crystal is known to be surrounded by {111} facets. As fcc {111} and hcp {001} have the same close-packed atomic arrangement, only a moderate lattice mismatch of 3.6% exists between Ru {001} and Pd {111}, which enables the relatively perfect epitaxial growth of a Ru {001} monolayer. Octahedrons with a size of about 40 ± 5 nm were observed by bright-filed TEM and HAADF-STEM (Figure 5(a) and (b)). The tip of the obtained octahedron is shown in the aberration-corrected high-resolution HAADF-STEM image in Figure 5(c). The lattice fringes of 0.225 and 0.195 nm correspond to the {111} and {200} planes of Pd, respectively. The aberration-corrected HAADF-STEM images clearly show a sharp color contrast and stack faults near the surface, indicating deposition of Ru atoms on the Pd surface (Figure 5(d) and 5(e)). The formation of stack faults may result from the fcc-to-hcp phase transition near the interface area. The interplanar distance between the topmost and second layer was 2.53 Å (Figure 5(e)), which was significantly larger than the interlayer distance of Ru (2.15 Å) or Pd (2.24 Å), indicating a monolayer rather than a bilayer of Ru atoms. The unique porous structure with trace amounts of Ru on the Pd surface strongly enhanced the activity and selectivity towards semihydrogenation of various alkynes including phenylacetylene (Figure 6(a) and (b)) and methyl non-2-ynoate (Figure 6(c) and (d)). In particular, for the semihydrogenation of internal alkyne (i.e. methyl non-2-ynoate) reaction, the porous Pd octahedra covered with monolayer Ru atoms exhibited >99% conversion efficiency and 92% stereo-selectivity in 1,2(*Z*)-alkene. However, without the Ru monolayer, the porous Pd octahedra only had 56% conversion and near 60% stereo-selectivity in 1,2(*Z*)-alkene. The specific activity was five times higher than that of original Pd octahedrons. After four cycles, no change was observed in both activity and stereo-selectivity. The authors ascribed such good selectivity to the synergistic effects between monolayer Ru and porous Pd. Because ruthenium catalysts are ready to form stable intermediates in the hydrogenation of alkynes, the stable intermediates adsorb firmly on the Ru catalysts surface and block the decomposition of surface active sites, which may be beneficial to the selectivity as well as stability.

Most recently, Ye and co-authors synthesized a Ru fcc octahedral nanoframe.[71] The fcc Ru nanoframe was evolved from Pd@Ru core-shell structure with a 2-nm shell thickness. Similar with the abovementioned methods, a two-step seeded growth process was applied. The Ru atoms were deposited on the surface of preformed Pd octahedrons in EG solution at 200 °C. After etching the Pd core by Fe^3+^/Br^-^ pair at 90 °C, the Ru nanoframe was obtained. However, the molar ratio of Ru to Pd in Ru nanoframe was determined to be ∼4.5:1 by inductively coupled plasma-optical emission spectrometry (ICP-OES). The authors indicated that a small portion of Pd may exist in Ru nanoframes in the form of Ru/Pd alloy because of the interdiffusion between Ru and Pd at high temperature.

To date, all the reported core-shell structures are Ru shell on Pd core, and the inverse architecture has not yet been reported.

## 5. Solid-solution type Pd–Ru NPs

Compared with the above-mentioned phase-separated nanoalloys, solid-solution type nanoalloys have two noteworthy advantages arising from alloying effects. One is related to the atomic and/or geometric configuration on the surface of bimetallic nanomaterials. In contrast to the core–shell structure, whose surface is covered with only one element, two elements are randomly or orderly distributed on the surface of solid-solution alloy NPs. Therefore, the bifunctional effect, which describes that both components take part in the reaction, is proposed to account for the enhancement of catalytic properties. One of the most outstanding examples is Pt–Ru solid-solution alloys for methanol electrooxidation reaction (MOR).[47,72–74] It is known that active sites of pure Pt catalysts are occupied by the chemisorbed CO intermediate during MOR. Such ‘poisoning effect’ of chemisorbed CO deteriorates the catalytic performance of Pt in MOR. Alloying Pt with other transition metals is one of the best-known remedies for this poisoning effect, and Ru appears to be one of the best promoters among the transition metals. Two mechanisms have been proposed for the promoter effect of Ru. One is electronic effect by alloying which will be discussed later. The other is the bifunctional mechanism. In the bifunctional mechanism, the surface Ru atoms will provide oxygen-containing species by activating water at more negative potentials than Pt, and the oxygen-containing species will accelerate the CO oxidation on Pt sites.[47] The realization of the bifunctional effect, however, requires specific surface atom configuration.

The other advantage is more prominent. In solid-solution alloy NPs, two kinds of metal atoms are distributed randomly or orderly, which means that the electronic state of materials can be continuously controlled by tuning the compositions. The properties of materials, particularly solids, directly depend on their electronic states, e.g. their density of states (DOS) at the Fermi level. Taking the Pt–Ru solid-solution NPs for MOR as an example again, in the electronic effect mechanism, it is believed that the total DOS at *E*_F_ will downshift by electron donation from Pt to Ru and thus the interaction between Pt and CO becomes much weaker.[72,74] Another outstanding example is the Ag–Rh solid-solution alloy NPs reported by our group in 2010.[36] Ag and Rh are two neighbor elements of Pd. Although the bulk elements Rh and Ag demonstrate attractive properties towards many applications, they cannot store hydrogen under ambient conditions. Pd is the only element that can absorb hydrogen under ambient conditions among late transition metals. Surprisingly, we found that the Ag_0.5_Rh_0.5_ solid-solution alloy was capable of absorbing hydrogen. The hard X-ray photoelectron spectroscopy (HAXPES) indicated that the electronic structures around the Fermi energy were very similar between Ag_0.5_Rh_0.5_ alloy NPs and Pd NPs,[75] indicating that Ag_0.5_Rh_0.5_ solid-solution NPs can be considered as ‘artificial Pd’. Such a result was also supported by first principles calculation. This example well demonstrated the concept of ‘DOS engineering’.[76] However, it is worth noting that a definitive determination of the alloying effect remains elusive. For a target application, a rational synthesis of Pd–Ru solid-solution alloy NPs with controlled composition is required.

Motivated by the Ag–Rh case, we were the first to synthesize the Pd_x_Ru_1−x_ (0 < x < 1) solid-solution alloy NPs in 2014.[50] Ru and Pd are two neighbor elements of Rh. Rh is highly active towards various reactions especially in automotive and industrial exhaust gas treatment.[77] However, as Rh is one of the most expensive metals because of its scarcity, we had to use this element efficiently. The Ag–Rh case caused the speculation that the Pd_0.5_Ru_0.5_ solid-solution alloy NPs may have similar electronic state and properties to Rh. Thus, Pd–Ru solid-solution NPs are considered a potential alternative to Rh.

Pd_x_Ru_1−x_ solid-solution NPs were obtained by a wet-chemical synthesis based on a modified polyol method. In the traditional polyol methods, metal NPs are synthesized by heating the metal precursors solution from r.t. to a desired temperature. However, for the Pd–Ru case, a mixture of monometallic NPs would be obtained under the traditional process because of the large difference in reduction kinetics and the big miscibility gap between Pd and Ru. Taking triethylene glycol (TEG) as an example of a reductant, Pd would burst into nucleation and grow fast into large nanocrystals around 80 °C. Ru ions would not be reduced quickly until 170–180 °C. In our modified polyol method, a mixture of metal precursors (K_2_PdCl_4_ and RuCl_3_·nH_2_O) was added simultaneously and slowly into a preheated TEG solution (200 °C) that contained polyvinylpyrrolidone (PVP) as a protecting agent. At such a high temperature, both Pd and Ru ions were simultaneously and rapidly reduced into zero-valence atoms with negligible difference in reduction speed.

**Figure 7.**
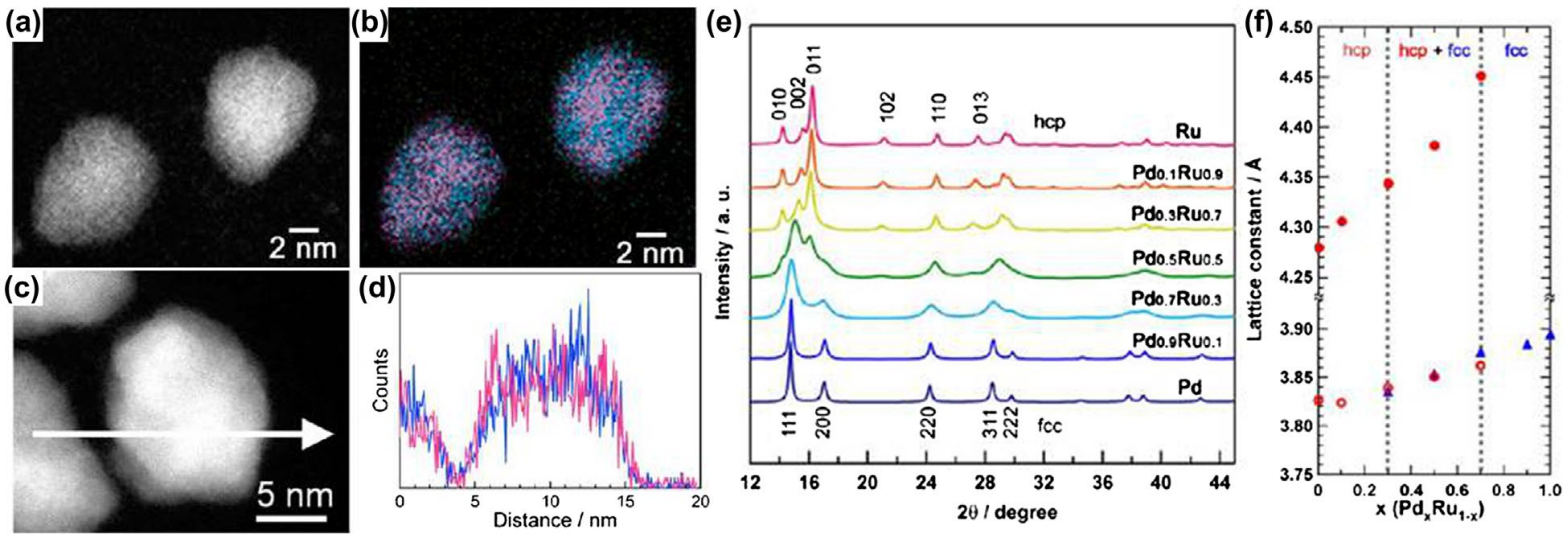
(a) and (c) HAADF-STEM images of Pd_0.5_Ru_0.5_ NPs, (b) the corresponding STEM-EDX mapping images and (d) compositional line profiles of Ru (red) and Pd (blue) of the NPs in (a) and (c) respectively. (e) Synchrotron powder XRD patterns of Pd_x_Ru_1−x_ NPs at 303 K (*λ* = 0.57803(2) Å). (f) Relationship between the lattice constants of Pd_x_Ru_1−x_ NPs obtained by Rietveld refinements and Pd content (x) in the NPs. The lattice constant of the fcc component (blue triangles), and the lattice constant of (a) (red open circles), and (c) (red closed circles) of the hcp component. (Reproduced with permission from [50] © 2014, American Chemical Society.)

The STEM-EDX mapping and compositional line profiles of optional Pd_0.5_Ru_0.5_ NPs demonstrated that Ru and Pd atoms were distributed homogenously, verifying the solid-solution nature (Figure 7(a)–(d)). The synchrotron powder XRD pattern of the Pd_x_Ru_1-x_ NPs is shown in Figure 7(e). With increasing Ru content, the crystal structure of the as-prepared Pd_x_Ru_1−x_ solid-solution NPs gradually changed from the Pd fcc to the Ru hcp lattice. The lattice constants of Pd_x_Ru_1-x_ NPs obtained by Rietveld refinement are shown in Figure 7(f). The lattice constants Pd_x_Ru_1-x_ NPs followed Vegard’s law, further suggesting the formation of solid-solution alloys structure in the whole composition range. In the Pd_x_Ru_1−x_ NPs (0.3 ≤ x ≤ 0.7), hcp and fcc structures coexisted. However, the coexisting hcp and fcc structure had approximately the same metal ratios. In addition, the metal ratios in each phase were consistent with the EDX data.

**Figure 8.**
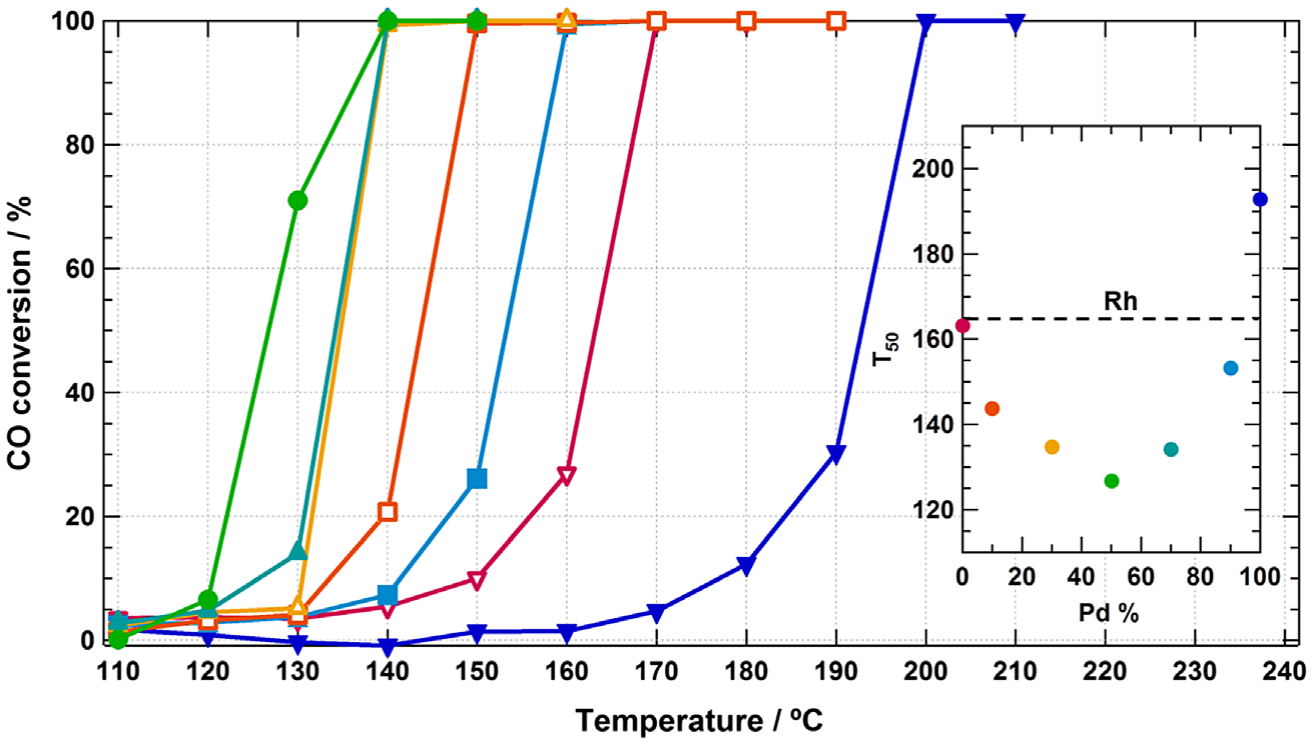
Temperature dependence of CO conversion in Pd_x_Ru_1−x_ nanoparticles supported on γ-Al_2_O_3_; x = 0 (∇ red), 0.1 (□ orange), 0.3 (Δ yellow), 0.5 (● green), 0.7 (▲ blue-green), 0.9 (■ light blue), and 1.0 (▼ blue). The inset is the metal-composition dependence of T_50_. The black dashed line represents T_50_ of Rh NPs. (Reproduced with permission from [50] © 2014, American Chemical Society.)

The Pd_x_Ru_1−x_ solid-solution NPs were loaded on γ-Al_2_O_3_ to check their catalytic performance for CO oxidation. Rh NPs and a physical mixture of Ru NPs and Pd NPs were used as control groups. The metal-composition dependence of the CO conversion curves in γ-Al_2_O_3_ supported Pd_x_Ru_1−x_ NPs is shown in Figure 8. In addition, the temperatures for 50% conversion of CO to CO_2_ (T_50_) of Pd_x_Ru_1−x_ NPs are shown in Figure 8 inset. All the Pd_x_Ru_1−x_ NPs exhibited higher catalytic activities than monometallic Pd or Ru NPs, and Pd_0.5_Ru_0.5_ NPs had the best activity. The XPS spectra indicated that the electron transferred continuously from Pd to Ru with increasing Ru content. It is believed that the d-band center of Pd_0.5_Ru_0.5_ became optimized for the absorption of O and CO, endowing lower activation energy for CO oxidation. Such an example strongly confirmed that the electronic structure of each constituent could be continuously controlled by changing the composition in the solid-solution type nanoalloy.

We further found that the Pd–Ru solid-solution alloy NPs also showed enhanced catalytic activity towards the Suzuki–Miyaura cross-coupling reaction.[78] Pd and its compounds are widely used as catalysts to afford C–C bond formation by the Suzuki–Miyaura cross-coupling reaction. However, Pd faces a leaching problem and gradually loses its activity.[78] By using supportless Pd–Ru solid-solution NPs instead of pure Pd NPs, we found that the former exhibited much higher selectivity and activity than either Pd or Ru NPs alone and the optimum combination for NPs was Pd_0.5_Ru_0.5_. Moreover, there was negligible metal leaching during the long cycle catalytic process. Such outstanding catalytic activity of Pd_0.5_Ru_0.5_ NPs was ascribed to electronic modification of the Pd and Ru elements by alloying. Specifically, on the surface of Pd_0.5_Ru_0.5_ NPs, uniform coexistence of both opposite charges (Pd^δ+^ and Ru^δ-^) can possess acceptor and donor properties as well as dual Lewis acid/base properties at the same time.[78] Additionally, the homogenous distribution of Pd and Ru atoms on the surface, which is vital for the catalysis, reflects the idea of a bifunctional effect to some extent. Very recently, we found that the Pd–Ru solid-solution NPs were highly active in the three-way catalytic (TWC) reaction, which is an important catalytic process simultaneously purifying harmful gasses including NO_x_, CO, and hydrocarbons in exhaust gas.[79] To our surprise, the Pd–Ru solid-solution alloy NPs outperform Rh NPs towards the TWC reaction. In comparison with monometallic Rh NPs, the electronic structure of the Pd–Ru alloy is considered to be similar to that of Rh. Therefore, the solid-solution alloy structure may provide a suitable reaction field for both reduction and oxidation reactions in such a complex TWC reaction system.

Another interesting catalytic reaction, formic acid oxidation (FAOR), was also performed on the Pd–Ru alloy system by Wu et al.[53] FAOR has been broadly studied as a model reaction from both practical and scientific viewpoints as an anodic reaction for direct formic acid fuel cells. It is considered that Pd is the most efficient metal catalyst towards FAOR.[80] However, pure Pd metal tends to dissolve in the acidic electrolyte during potential sweeping. To address this point, alloying with Pd has been adopted as a powerful strategy. To date, various Pd-based alloys have been synthesized for FAOR such as Pd–Cu and Pd–Co.[81] Although Ru does not show catalytic activity as high as Pd or Pt towards fuel cell anodic reactions, it serves as a promoter to Pd or Pt catalysts. The most well-known sample is Pt–Ru mentioned above; Ru does not participate the MOR directly, but Pt–Ru is one of the best catalysts for MOR.[45] However, there is no report about using Pd–Ru solid-solution nanoalloys for FAOR.

**Figure 9.**
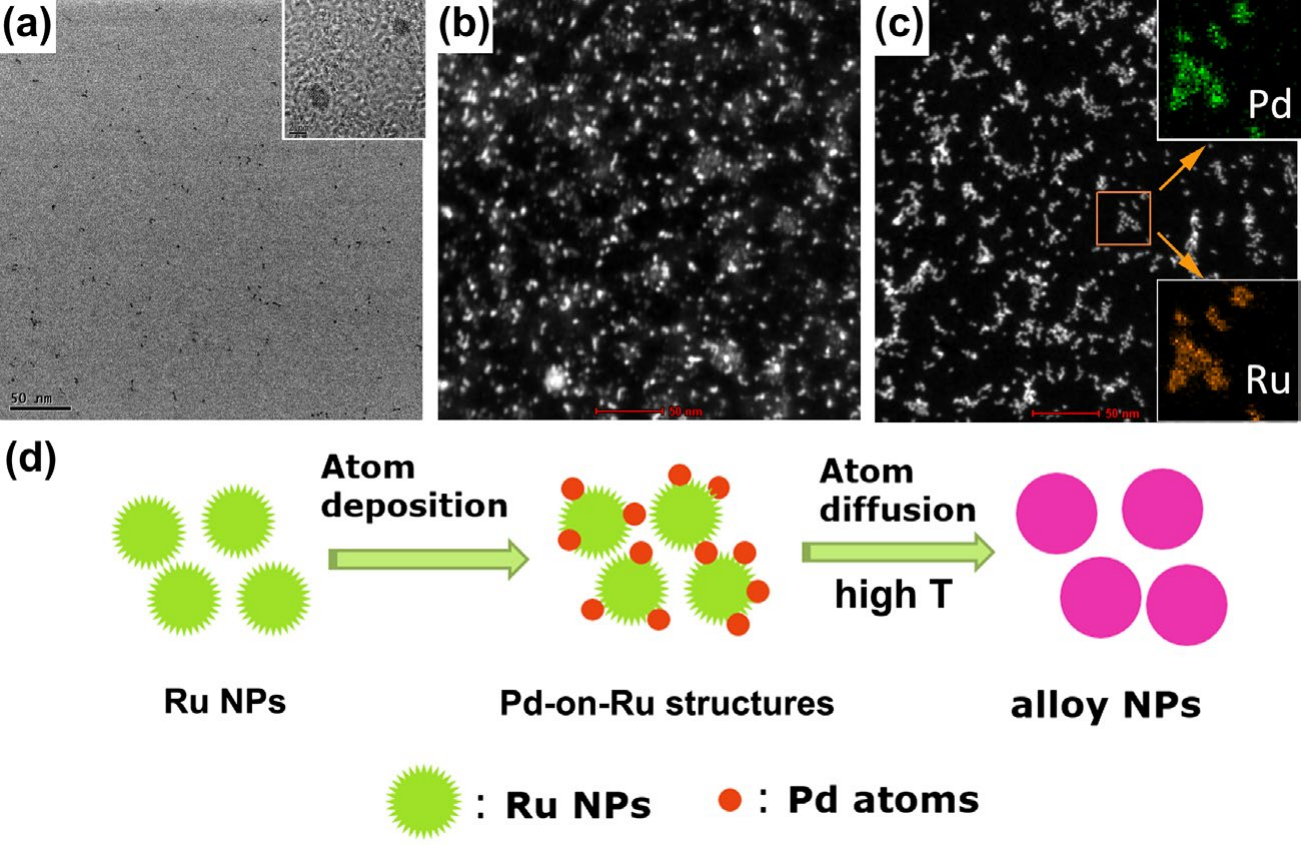
Time-dependent TEM/HAADF-STEM images taken during the formation of Pd_0.6_Ru_0.4_ NPs: (a) 40 s, the presence of Ru NPs, inset shows a HRTEM image, (b) 3 min, half of the volume of the K_2_PdCl_4_ solution was dropped to form heterostructures, (c) 5 min, finished adding K_2_PdCl_4_, the insets in 9c show the corresponding EDX mapping of the squared area, (d) proposed formation mechanism. (Reproduced with permission from [53] © 2014, Wiley.)

Wu et al. [53] developed sub-5 nm Pd_x_Ru_1−x_ solid-solution NPs with the whole composition range and investigated the composition-depended catalytic properties for FAOR^53^. Unlike the co-reduction method reported by us, the synthesis developed by Wu et al. is based on an atomic diffusion mechanism. In Wu’s method, Ru ions, which are more difficult to reduce than Pd ions were firstly added to a boiling EG solution and quickly formed disordered Ru clusters (Figure 9(a)). When the Pd precursor was introduced into the EG solution, the Pd atoms favored heteronucleation and were then deposited on the rough surface of the Ru clusters by forming a Pd-on-Ru heterostructure (Figure 9(b)). With further processing of the reaction under such a high temperature (close to the boiling point of EG), atomic diffusion would be energetically favored and lead to the formation of solid-solution alloy NPs (Figure 9(c)). STEM-EDX mapping and line scan confirmed the solid-solution nature (Figure 9(c) insets). Such atomic diffusion mechanism was illustrated in Figure 9(d).

**Figure 10.**
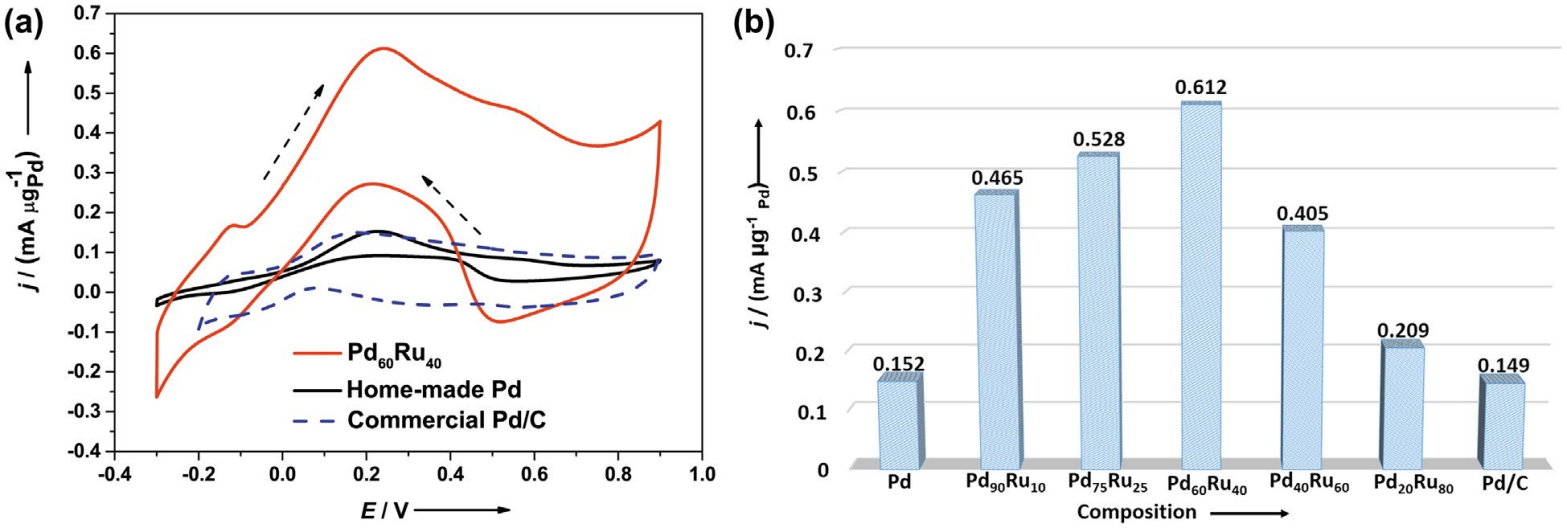
(a) The j-V curve in 0.1 M HClO_4_ and 0.25 M HCOOH solution at 50 mV s^−1^ on the Pd_60_Ru_40_ NPs/C, homemade Pd and commercial Pd/C catalysts. (b) Forward peak current density for formic acid oxidation as a function of Pd/Ru composition and commercial Pd/C is included. (Reproduced with permission from [53] © 2014, Wiley.)

The sub-5-nm Pd_x_Ru_1−x_ solid-solution NPs demonstrated a volcano-type behavior in the composition–activity relationship for FAOR. Although Ru does not have any catalytic activity towards FAOR, all the Pd_x_Ru_1−x_ solid-solution NPs showed higher activity than pure Pd NPs. Pd_0.6_Ru_0.4_ exhibited the optimum catalytic performance in terms of both activity and stability for FAOR (Figure 10 (stability not shown)). XPS spectra showed the electron transfer from Pd to Ru in sub-5-nm Pd_x_Ru_1−x_ solid-solution NPs, which is consistent with our report and the abovementioned near-surface Pd–Ru alloys studies.[50,58] This phenomenon clearly demonstrated the modification of electronic state of Pd by alloying Pd with Ru. Such electron transfer would promote the downshift of the d-band center, which endows the prepared Pd–Ru alloys with more active and stable catalytic properties for FAOR.

Presently, there are continuous reports on the use of Pd–Ru solid-solution alloys with specific compositions for numerous applications, such as methanol electrooxidation,[82] ethanol electrooxidation,[83,84] glycerol oxidation and detection,[85] hydrogen oxidation,[86,87] benzoic acid hydrogenation,[54] and phenol hydrogenation.[88] For instance, Tang et al. [54] have prepared an N-doped C-supported Ru–Pd solid-solution alloy with an average particle size of 3.6 nm by the ultrasound-assisted co-reduction method^54^. The prepared Ru–Pd solid-solution type nanoalloy showed a remarkably superior activity, stability and selectivity to their monometallic counterparts in the benzoic acid hydrogenation reaction. St. John and co-workers prepared carbon-supported Pd_x_Ru_1-x_ solid-solution alloys for the application of hydrogen electrooxidation (HOR) in alkaline solution.[87] They found that Pd_x_Ru_1-x_ solid-solution alloys showed an approximately two–threefold increase in the exchange current density over pure Pd catalysts. The reasons for this can be attributed to the reduction in the metal–hydrogen bond binding energy, which is the key point in the electron transfer rate-determining step for HOR.

The above-mentioned examples unambiguously show that the properties of Pd–Ru solid solutions are strongly enhanced because of the alloy effects brought by the electronic structure change or bifunctional mechanism.

## 6. Perspective

The surprising potential of nano science was first suggested in Richard Feynman’s 1959 talk titled ‘There’s plenty of room at the bottom’.[89] After half a century of development, new materials with unexpected properties have been found at the nanoscale. We reviewed the recent development of Pd–Ru bimetallic nanomaterials based on newly developed synthetic methods and analytical tools in nanoscience. We emphasized that the nanosize effect is a powerful tool to create novel nanomaterials. We stressed the importance of electronic structure modification by controlling the nanostructure including solid-solution, core-shell, and heterostructure to bring out attractive catalytic properties. Importantly, by using Pd–Ru solid-solution NPs as an example, we clearly showed the importance of the ‘DOS engineering’ concept in guiding the rational design of functional materials.

However, there are still many untouched topics in the study of the Pd–Ru bimetallic nanomaterials. For example, the size effect of Pd–Ru bimetallic nanoalloys has not yet been revealed. Recently, Yin and co-workers [90] studied the effects of the finite size in the electronic structure of ultrathin Pd (111) films grown on Ru (0001) by varying the thickness of atomic layers. They found that the Pd (111) films containing fewer than five monolayers were surprisingly inert towards oxygen despite the fact that bulk Pd (111) was highly reactive. Therefore, it is expected that the Pd–Ru bimetallic nanomaterials will exhibit size-dependent physical and chemical properties. In addition, with the size decreasing to nanoscale, the NP may adopt a crystal phase that is quite different from bulk. Our group reported the synthesis of fcc Ru NPs.[91] Therefore, phase control in Pd–Ru bimetallic nanomaterials becomes an interesting topic. Such works are currently being investigated.

Not limited just to the Pd–Ru bimetallic nanomaterials, we note that theoretical modeling becomes increasingly important in the prediction and/or explanation of properties of nanomaterials. Last but not least, the development of *in situ* experimental techniques for characterizing nanomaterials under working conditions, such as *in situ* TEM, ambient environment XPS, etc., are now highly desired. A rational design in combination with theoretical modeling and *in situ* observation will be a robust way to effectively prepare nanomaterials for special applications.

## Disclosure statement

No potential conflict of interest was reported by the authors.

## Funding

This work was supported in part by the JST CREST and ACCEL programs. The authors declare no financial competence.
